# The Back Pain and Movement (B-PAM) registry; a study protocol

**DOI:** 10.1186/s12891-019-2625-x

**Published:** 2019-05-24

**Authors:** Mehul J. Desai, Holly Jonely, Meagan Blackburn, Sangeeth Wanasinghage, Sidrah Sheikh, Rod S. Taylor

**Affiliations:** 1 International Spine, Pain & Performance Center, 2141 K Street NW, Suite 600, Washington, DC 20037 USA; 2 The George Washington University, School of Medicine and Health Sciences, 2300 I St NW, Washington, DC 20052 USA; 3 dorsaVi Ltd, 86 Denmark Street, Kew, Victoria 3101 Australia; 4 University of Exeter Medical School, St Luke’s Campus, Heavitree Road, Exeter, EX1 2LU UK

**Keywords:** Low Back pain, Registry, Technology, Movement, Inertial measurement unit

## Abstract

**Background:**

Low back pain (LBP) is a ubiquitous, heterogeneous disorder that affects most people at some point in their lives. The efficient management of LBP remains elusive, with direct and indirect costs attributed to LBP surpassing many other common conditions. An emphasis on a structural basis of LBP often fails to recognize movement, specifically patterns of movement that may provide biomechanical signatures of painful conditions. The primary objective of this registry is to understand the differences in movement patterns among those with LBP and those without pain in a U.S. population sample.

**Methods:**

This ongoing, non-randomized, prospective post-market registry will consist of two groups: patients with LBP, and age and sex-matched controls without LBP. We will seek to recruit 132 subjects in each group. Data collection will take place in two phases: (1) baseline assessment of LBP patients and matched controls; (2) assessment of LBP patients at 6 and 12-months follow up. The primary outcome measure will be differences in movement patterns between those with LBP and those without LBP. Secondary outcomes will include differences in patient reported outcomes including pain, disability and quality of life.

**Discussion:**

The findings will help determine if there are meaningful differences in movement patterns between those with and those without LBP. Further, an initial understanding of movement signatures specific to certain subtypes of patients with LBP may be achieved.

**Trial registration:**

The study was registered on the clinicaltrials.gov portal: NCT03001037. Trial retrospectively registered 12/22/2016.

## Background

Low back pain (LBP) represents a complex constellation of conditions that commonly afflicts the adult population. As the main contributor to years lived with disability [1], it is an enormous public health issue, with associated expenditures being three times greater than diabetes, cancer and heart disease [2]. Despite these costs, the treatment and prognosis for persistent LBP remain quite poor. Unfortunately, symptomatic mismanagement of LBP likely represents an underlying lack of understanding of the multifactorial contributions to the condition. Importantly, dynamic real-time understanding of movement patterns and biomechanics have historically been rudimentary and are often dependent upon the skill and expertise of the examiner. Moreover, assessments of these key factors frequently rely on the “eyes” of the clinician and the subjective feedback of the patient.

A systematic review and meta-analysis of studies comparing lumbo-pelvic kinematics in people with and without back pain demonstrated significant limitations in the included studies, specifically a lack of detail or standardization between studies on the criteria used to define participants as those with LBP or without LBP [3]. Furthermore, the reliability of the most common means of measuring lumbar sagittal motion (plurimeter V double inclinometer, the carpenter double inclinometer and the computerized single sensor inclinometer) was found to be greatly varied, falling below clinically desirable levels with errors primarily attributed to the examiner [4]. Using 3D lab-based motion analysis, Hemming et al. [5] evaluated spinal kinematics between healthy individuals and subjects with chronic low-back pain. They observed significant differences in lower-thoracic and upper-lumbar movement during functional tasks in a UK-based population. However, they specified a lack of analysis of muscle activity as a limitation to better understand underlying pain mechanisms. Moreover, they noted that use of continuous postural measurement devices may improve understanding around movement-related behavior, and behavioral change [5].

Recent technological advances offer new opportunities for accurate, convenient measurement of movement and posture as correlated to pain, thus theoretically allowing a more in-depth understanding of the foundations of chronic LBP. Laird et al. [6] utilized wireless, wearable, sensor technology (ViMove; dorsaVi Ltd., AUS) to evaluate lumbo-pelvic kinematic patterns during sagittal flexion movements. They identified four subgroups which distinctly differ in range and speed of motion, muscle relaxation, and lumbo-pelvic contribution to movement. These findings demonstrated the feasibility of subgrouping without pre-classification based on observation or subject history and highlighted the heterogeneity of lumbo-pelvic kinematics within an LBP sample. They also support the notion of improving treatment efficacy by tailoring to kinematic deficits as opposed to subjective pain experiences or observations.

It is of note that sagittal flexion was the only evaluated movement, with no inclusion of other single-plane or multi-plane movements. As such, functional movement-related classifications described by Hemming et al. [5] were not incorporated. Similarly, O’Sullivan [7] described specific classifications which manifest only during functional tasks. However, the study was limited to an Australian population, and as discussed by the authors, results have not been verified in an independent sample.

Matheve et al. [8] assessed the effectiveness of sensor-based postural feedback in modifying lumbo-pelvic motor control in Belgian patients with chronic LBP, and healthy controls. The intervention comprised of two movement control tasks conducted in a single exercise session (lifting task & waiter’s bow). The authors concluded that postural feedback was effective in improving lumbo-pelvic control. Tsang et al. [9] evaluated the difference in lumbo-pelvic movement patterns between healthy and low back pain subjects using a 3-dimensional inertial sensor system (3D MyoMotion). Seventeen males with low back pain and eighteen males without were instructed to bend forward at five selected speeds while wearing the sensors. The study evidenced the system’s ability to track movement patterns and highlighted that subjects with low back pain adapt to carry out the same motion as those who are asymptomatic.

Other studies of posture and back movements combined inertial sensors with electromyography (EMG) sensors. In a preliminary study of the mDurance device, Banos et al. [10] conducted trunk endurance assessments on subjects currently undergoing physical therapy. The device was effectively able to manage movement data recorded during the tests. In a cross-sectional study by Laird et al. [11] used the combined motion and surface electromyography (sEMG) capabilities of ViMove and observed diminished range of motion in the trunk, lumbar, and pelvis along with slowed overall movement in those with low back pain. They also noted significant differences of flexion-relaxation between LBP and healthy controls as assessed through sEMG. However, the study was limited to evaluation of flexion and seated postures, and only examined univariate relationships.

To better understand and characterize movement patterns both in populations with and without LBP in a broader set of movements, dorsaVi established the Back Pain and Movement (B-PAM) Registry. The purpose of B-PAM is to provide baseline, follow-up, and normative data pertaining to single-plane and dynamic movements of normal controls as well as those with LBP, as assessed by ViMove, in a U.S. sample population. ViMove is capable of continuously measuring 3-dimensional lumbosacral movements and sEMG for up to 24 h. This system has been validated against gold standards for motion analysis including Optotrak and Vicon motion capture systems where it demonstrated clinically acceptable agreement with these motion laboratory-based systems [12]. The movement consistency of ViMove between tests on the same and different days has also been evaluated for flexion, extension, and lateral flexion [13]. Intraclass correlations of r = .88 (range .80 to .98) and r = .85 (range .69 to .97) were observed respectively indicating good to excellent agreement. ViMove has received FDA 510(k) clearance and is commercially available.

By extending on previous research which only focused on certain movements, and by additionally evaluating sEMG, B-PAM seeks to provide an extensive repository of data for use in defining assessment and treatment algorithms while evaluating performance of specific movements. The B-PAM data is intended to benefit and support interests of patients, practices, hospitals, clinicians, regulatory bodies, payers and industry by streamlining the clinical surveillance process and facilitating leading edge performance assessment while also gathering baseline data without intrusion into clinical pathways.

The registry seeks to address the following questions in a U.S. population sample:

1. Are there differences in range of movement and kinematics (e.g. speed and pelvic/lumbar contribution of movement) between patients with LBP and subjects without LBP?
2. Are there differences in secondary outcomes (leg/back pain, depression, health-related quality of life) between those with LBP and without LBP?
3. Are there identifiable lumbo-kinematic patterns and muscle-activity associated with LBP patients during single-plane and dynamic movement?
4. Are there associations between the demographic and clinical characteristics (e.g. age, gender, level of back pain) and kinematic outcomes in LBP patients?

## Methods/design

This is an ongoing, non-randomized, prospective post-market registry consisting of two groups: patients with LBP, and age and sex-matched controls without LBP. We will recruit a total of 132 subjects in each group. B-PAM has a flexible design to allow new dorsaVi products to be added to the registry following their market release. Data collection will take place in two phases: (1) baseline assessment of LBP patients and matched controls; (2) assessment of LBP patients at 6 and 12-months follow up.

Reporting and data analysis of the B-PAM registry will be undertaken in accord with The Strengthening the Reporting of Observational Studies in Epidemiology (STROBE) guidelines for reporting observational studies [14].

### Site selection

Sites participating in B-PAM will initially be two clinical centers in Washington DC, USA (International Spine Pain & Performance Center and Synergy Manual Physical Therapy).

### Subject selection

Subjects with LBP will be included if they fulfil the following criteria:

- Subject or legally authorized representative provides written authorization and/or consent per institution and geographical requirements
- Subject with a predominant complaint of LBP of any duration, with a minimum daily pain average visual analogue score (VAS) ≥ 30/100
- Subject is intended to be assessed with the eligible product

Exclusions to enrollment are:

- Unable to be available for study follow-ups
- Exclusion criteria required by local law
- Movement assessment is contraindicated for any reason.
- Currently enrolled in or plans to enroll in any concurrent drug and/or device study that may confound results
- Body mass index ≥35 kg/m^2^

Controls will be recruited using study advertisement flyers posted on campus at the George Washington University, local gymnasiums, recreational centers, via word of mouth, and email list servers associated with educational programs within The George Washington University School of Medicine and Health Sciences. Subjects will be screened by phone for the presence or history of LBP (no current LBP and no history of LBP lasting longer than 3 months in the past 12 months) as well as other exclusion criteria listed above. Controls will be age and sex-matched with LBP patients on a one-to-one basis.

### Minimization of bias

The following procedures have been incorporated to minimize potential bias:

- Sites will screen and consecutively enroll all subjects who undergo assessment.
- The Principal Investigator will be blinded to results of assessment.
- Sites must meet pre-defined criteria to be selected to participate.
- Standard operating procedures for data collection will be employed across study sites with a single common database for all sites.

### Sample size

Detection of a ‘moderate’ effect size (i.e. Cohen’s effect size of ≥0.40 in range of movement and other outcomes) between LBP and controls requires 132 individuals per group at 90% power and alpha of 5% (estimated using STATA’s sampsi command) [15].

Based on this sample size and using data in individuals with no back pain reported by Laird (Monash University, Research Project Report No: cf11/0748–2,011,000,372), Table 1 shows the predicted minimum mean difference that could be detected between back pain and control groups. The magnitude of these predicted between-group differences in range of movement appear to be fairly consistent with those reported in the meta-analysis conducted by Laird et al., evaluating previously published studies comparing LBP and control populations [3].

**Table 1:** Range of motion and predicted minimum mean differences between back pain and control groups

ROM outcome	Non-back pain group Observed Mean (SD)	Predicted minimum mean difference between control and back pain group
Lordosis		
30–39 years	-30 (6.9)	± 2.76
40–49 years	−32 (10.1)	± 4.04
50–65 years	− 36 (12.2)	± 4.88
Standing flexion		
30–39 years	46 (11)	± 4.4
40–49 years	48 (6)	± 2.4
50–65 years	46 (9)	± 3.6
Standing extension		
30–39 years	−26 (10)	± 4.0
40–49 years	−18 (11)	± 4.4
50–65 years	−17 (14)	± 5.6

We have also estimated the impact sample size of 132 back pain individuals in terms of achieving our second research objective of assessing the subject demographic and clinical characteristics associated with range of movement. Based on the rule of thumb of 10 observations needed per covariate (or predictor) [16], 32 individuals with back pain would allow for a multivariable regression including up to 13 covariates. 132 subjects would also allow detection of an increase in the f2 of multivariable model with 9 covariates from 50 to 55% with the inclusion in the model of one additional covariate at 90% power and 5% alpha (using STATA’s powerreg command) [17].

### Ethical approval

Prior to enrolling any subjects into B-PAM, sites must fulfil all local law and regulatory requirements. The term Ethics Board will be used to define the Institutional Review Board (IRB), Medical Ethics Committee (MEC), Research Ethics Board (REB) or Human Research Ethics Committee (HREC). Participation readiness includes but is not limited to:

- Ethics Board approval or a written statement by the Ethics Board Chairperson stating that approval is not required.
- Approval of the Informed Consent (CF) or Data Release Form (DRF) by the Ethics Board and/or sponsor (if required).
- A legally executed Agreement.
- Insurance certificates (as required by geography).
- Applicable Regulatory Approval (as required by geography).
- Documented training; signed and dated.

Subjects can exit from B-PAM if: they choose to withdraw, reach end of their follow-up period, the investigator deems withdrawal is necessary (e.g. medically justified, failure of subject to maintain adequate registry compliance, or if the subject is no longer available for follow-up.

### Measurements

General data collection procedures and the scope and timing of demographic data outcome collection are summarized in Tables 2 and 3 respectively.

**Table 2:** Summary of data collection

	Enrollment	Baseline/Assessment	Follow-Up	Exit
CF or DRF Signed and Dated	X	X		
Subject Demographics	X	X	X	X
Outcome Measures	X	X	X	X
Medical History	X			
Assessment Details	X	X	X	
Patient Status	X	X	X	X
Events	X	X	X	X
Protocol Deviations	X	X	X	X

**Table 3:** Summary of collected demographic and outcome data

Demographic measures	LBP Patients	Controls
Age	X	X
Gender	X	X
Pain episode duration (weeks)	X	X
Body Mass Index (BMI)	X	X
Employment (working vs not working)	X	X
Marital Status	X	X
Co-morbidities (categories)	X	X
Co-interventions - current medications (categories)	X	X
Co-interventions - (hospital admissions, doctor or other clinician visits, imaging, other diagnostic tests,	X	X
Patient outcomes		
LBP diagnosis (ICD-10) - clinician	X	
LBP – movement pattern classification (clinician)	X	
LBP – Movement Classifier	X	
QVAS - back	X	X
QVAS - leg	X	X
Oswestry Disability Index (ODI)	X	
START-Keele	X	
Fear Avoidance Behavior Questionnaire (FABQ)	X	
EQ-5D	X	X
Depression Anxiety Stress Survey 21 (DASS-21)	X	X
Patient perception of movement contribution to pain - single item	X	
Movement outcomes		
ViMove standard assessment (includes range of motion, speed, and timing of movement as described in “Measurements”)	X	X
30 s sit-to-stand test	X	X
40 m walk test	X	X

The dorsaVi ViMove system consists of two wireless tri-axial inertial measurement sensors containing accelerometers, gyroscopes, and magnetometers. These allow evaluation of movement in all three anatomical planes. Additionally, two wireless, bipolar, ViMove surface electromyography (sEMG) sensors will be placed on the subject’s low back via a disposable adhesive safe for human-use at standardized locations based on anatomy and subject height. Once these sensors are placed (at approximately the L3-vertebrae), the subject will be asked to move through a sequence of assessments (see Table 4).

**Table 4:** ViMove (dorsaVi) Assessment

Posture / Movement performed by subject:	Instructions to the Subject
Standing – ‘normal’ posture (calibration)	● Warm up – walk at comfortable pace for 20 s. ● Stop and reach towards the ceiling as high as you can. ● Stand with feet hip distance apart. ● Look straight ahead. ● Now we are going to measure your posture ● Stand still whilst I count to 5.
Flexion (*subject chooses speed, using support for balance if required)*	We want to see how you bend forward. ● When I say go, bend forward as far as possible, hold for 3 s, and then return to the starting position. ● Repeat this × 3
Extension	We want to see how you bend backward. ● Cross your arms over your chest. ● When I say go, bend back as far as possible and then return to the starting position. ● Repeat this × 3
Lateral flexion - right	We want to see how you bend sideways. ● Place your arms by your side. ● When I say go, slide your *right arm* down your right leg as far as possible and then return to starting position. ● Repeat this × 3
Lateral flexion - left	We want to see how you bend sideways. ● When I say go, slide your *left arm* down your left leg as far as possible and then return to starting position. ● Repeat this × 3
Pelvic tilt in standing	We want to see how you move your pelvis and see if you can move it without moving the rest of your body. ● This is how you tip your pelvis forward (demo) and this is how you tip your pelvis back. One forward and one back counts as one total movement. Have a practice (max 10 s) and I will start recording your movement. ● I want you to do this at least 3 times.
Usual sitting posture (as demonstrated by subject)	Take a seat in your chair. Show us how you usually sit.
Poor sitting posture (as demonstrated by subject)	Now show us how you sit when you are sitting poorly.
Good sitting posture (as demonstrated by subject)	Now show us how you sit when you are sitting in a good position.
Pelvic tilt in sitting – through full range	We want to see how you move your pelvis in the chair and see if you can move it without moving the rest of your body. ● Sit to the front of the chair. ● Roll forward as far as possible and then roll back onto your Tailbone as far as possible. This is how you tip your pelvis forward (demo) and this is how you tip your pelvis back. Have a practice (max 10 s) and I will start recording your movement. ● I want you to do this at least 3 times.
40 m (4x10m) Fast Paced Walk Test	“*For this test, do the best you can by going as fast as you can, without running, but don’t push yourself to a point of overexertion or beyond what you think is safe for you*. *1. Start with both feet on the start line.* *2. On start, walk as quickly but as safely as possible, without running.* *3. Walk up to the end cone, turn around and walk back to the starting cone behind you, turn again and back to the end cone, then turn once more and return to the start cone again so that you walk the 10 m walkway 4 times in total.* *4. Get ready and START”.*
30 s sit-to-stand test	“*For this test, do the best you can by going as fast as you can but don’t push yourself to a point of overexertion or beyond what you think is safe for you*. *1. Place your hands on the opposite shoulder so that your arms are crossed at the wrists and held close across your chest. Keep your arms in this position for the test.* *2. Keep your feet flat on the floor and at shoulder width apart.* *3. On the signal to begin, stand up to a full stand position and then sit back down again so as your bottom fully touches the seat.* *4. Keep going for 30 s and until I say stop.* *5. Get ready and START”.*

Motion sensor data will evaluate range of motion, acceleration, and velocity of movement, lumbo-pelvic contribution to movement, and onset of lumbar/pelvic movement.

- Contribution of pelvis vs. lumbar movement is calculated as the peak lumbar angle divided by trunk peak angle at end-range trunk flexion. This is evaluated in the movement ‘Flexion’ as defined in Table 4.
- Onset of lumbar/pelvic movement is similarly evaluated in the ‘Flexion’ task. Start of the flexion was defined as the point at which the velocity of movement exceeds 7°/s. This definition is automatically detected by the ViMove software.

Motion data is automatically processed by ViMove as follows:

- Accelerometer and gyroscope readings are low-pass filtered with a zero-phase, second-order Butterworth filter, and cut-off frequency of 5 Hz [18]. Data is sampled at 20 Hz.

sEMG data is automatically processed by ViMove as follows:

- Data is sampled at a frequency of 300 Hz
- A Band-Pass Filter is applied at 20 − 300 Hz
  - High-Pass Filter set to 20 Hz to reduce baseline drift and ECG components
  - Low-Pass Filter set to 300 Hz to create an envelope of the signal which is then anti-aliased and down-sampled.
- This signal is then used to calculate a moving root-mean-square average.

**Fig. 1:**
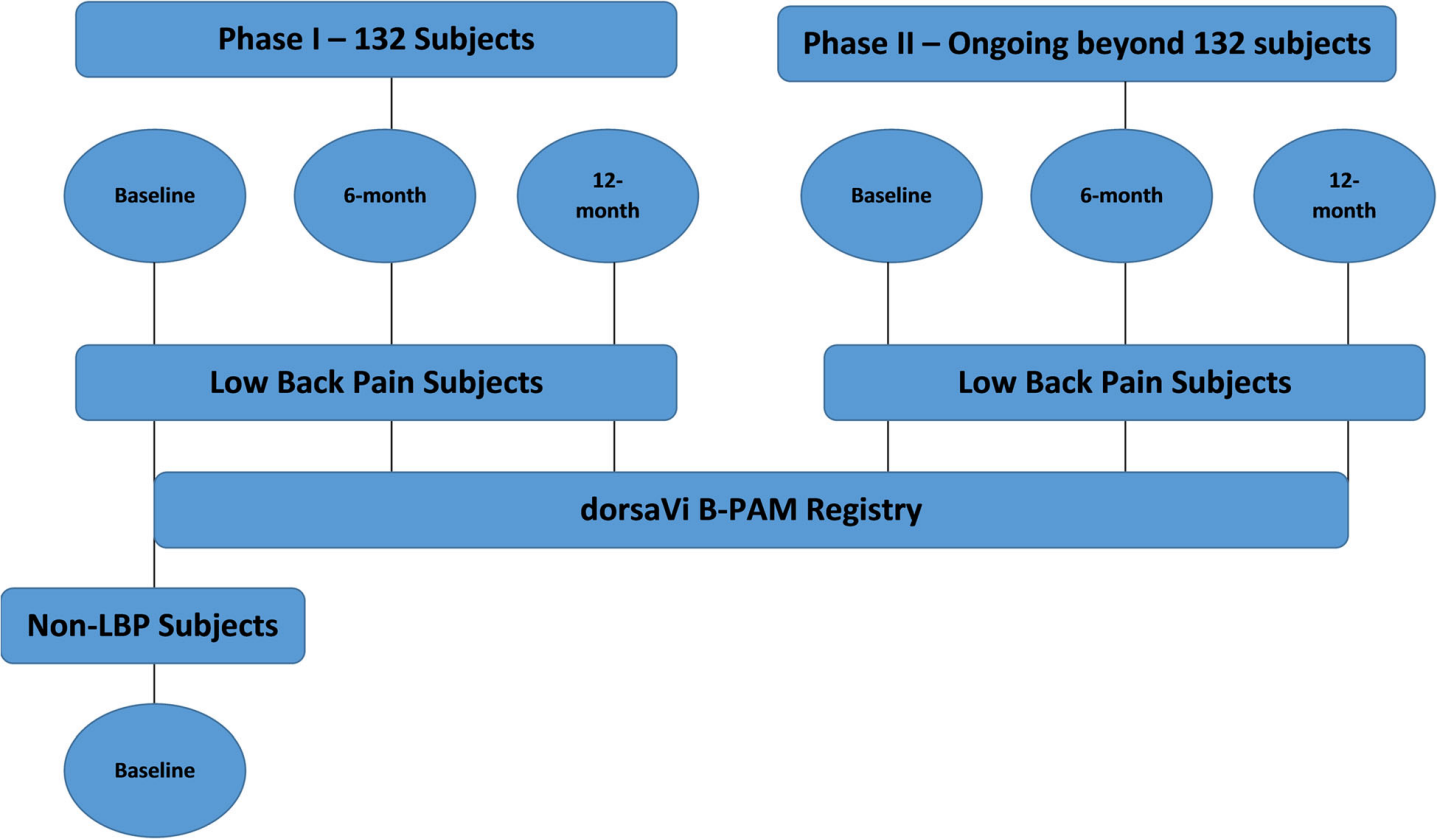
Schematic of study design

LBP patients and non-LBP controls will be assessed at enrolment (baseline). LBP patients will then then be followed up at 6 and 12 months (see Fig. 1).

### Statistical analysis

Baseline characteristics of LBP and controls will be reported descriptively as means (and standard deviations) or medians (and inter-quartile ranges) for continuous variables, or frequencies (and percentages) for binary variables. Groups will be compared using the independent 2-group t-test, Mann-Whitney U test, or Chi^2^–test respectively.

#### Comparison of range of movement and secondary outcomes between LBP subjects and non-LBP controls

Continuous primary and secondary outcomes will be compared between LBP and control groups using linear regression methods, and categorical outcomes will be compared using logistic regression. Models will be adjusted for baseline outcome scores (ANCOVA) plus matching variables and other characteristics found to be significantly different between groups at baseline. Between-group comparisons will be undertaken at baseline and each follow up. Within group differences in outcomes between baseline and follow up will also be examined. Residual plots will be used to check goodness of fit of regression models and, where necessary, power transformation of outcomes undertaken to improve model fit.

#### Association between demographic and clinical characteristics and range of movement outcomes in LBP subjects

Pearson correlation coefficients will be used to assess the univariable association between range of movement and various back pain subject characteristics. Multivariable associations will also be assessed using multivariable Pearson correlation coefficients and multivariate linear regression analysis. In determining multivariable models, collinearity between potential variables will first be assessed and variables where high collinearity will be excluded.

The focus on all data analyses will be on those registry participants with complete data. However, we will report rates and reasons for missing data and, where appropriate, consider imputation of missing cases using appropriate statistical methods.

All inferential analysis results will be reported as mean differences and 95% confidence intervals. We will report *P*-values to three decimal places with P-values less than 0.001 reported as *p* < 0.001. All statistical tests will be performed using two-sided tests at the 0.05 level of significance. No formal adjustment of the level of significance for testing will occur, but P-values will be interpreted accordingly, considering the multiple testing outcomes. An analysis will be undertaken at the end of the phase I, upon completion of enrolment of the age and sex-matched cohorts, prior to proceeding with enrolment of phase II of the registry.

All analyses will be undertaken using STATA version 14 [17].

### Data and quality management

Data will be collected using an electronic data management system and hard copy forms. Data reporting will be completed and submitted by the investigator or authorized staff. All data will be stored in a secure, password-protected database with a hard-copy backup in a locked file cabinet. Access will be controlled as delegated by dorsaVi to either a contract research organization (CRO) or to the principal investigator’s clinical site. This will allow for independent management of the data, which will be blinded to the investigators. The database will be backed up regularly.

The role of the CRO or Principal Investigator’s site would include data checking, data cleaning and data scoring. Ultimately, a scored/cleaned data set would then come to the study statistician to run the actual data analyses, further this data set would be provided to dorsaVi. dorsaVi may delegate an entity (CRO) to review site reported data to monitor quality. Data discrepancies will be highlighted as required and forwarded to the site for resolution. Site personnel are responsible for the timely submission or data and the resolution of discrepancies per their standard of care practices.

## Discussion

The Back Pain and Movement (B-PAM) registry provides the infrastructure to utilize the dorsaVi system to objectively measure movement and muscle activity in order to better understand movement patterns in LBP and non-LBP control populations. Furthermore, movement signatures among cohorts with similar diagnoses may be identified.

### Back pain registries

There are several ongoing registries that collect similar data. The Back-Pain Outcomes Using Longitudinal Data (BOLD) project specifically focused on those older than 65 with a new episode of LBP. This registry collects data including diagnostic tests, current treatments, pain, health-related quality of life and functional disability. Spine IQ is a registry specifically focused on performance measures. Designed to describe the natural history of disease, determine the clinical effectiveness of health care services and to measure/monitor safety and quality, it is focused on assisting U.S. clinicians with the transition to value-based care as mandated by U.S. legislation. Neither registry seems to focus on a deeper understanding of movement and its contributions to LBP. Furthermore, understanding patterns of movement as they relate to specific diagnoses are not emphasized.

### Strengths and limitations

Registries have the advantage that they enable easier patient enrolment than intervention trials such that large sample sizes are feasible. This increases the external validity, generalizability, and real-world applicability of data collected in registries. The non-observational nature of registries means that they are subject to confounding, selection bias, and poor data quality control. We have sought to minimize these limitations in B-PAM by use of age and sex matching of LBP controls, and through rigorous application of measures to ensure data quality.

### Future plans

We plan to extend the recruitment to additional US sites at a later date based on the following criteria:

- Clinicians familiar with and representative of the dorsaVi product;
- A patient population with more than 50 new LBP patients visiting monthly;
- Sites with adequate resources, facilities, equipment and support staff;
- Site is able to comply with registry requirements as well as local laws and regulations and dorsaVi requirements.

## Abbreviations

B-PAM: Back Pain and Movement Registry
DASS: Depression, Anxiety and Stress Scale
EQ-5D: EuroQol – 5D
FABQ: Fear-Avoidance Beliefs Questionnaire
ICD-10: International Classifications of Disease – 10
LBP: Low-Back Pain
ODI: Oswestry Disability Index
QVAS: Quadruple Visual Analogue Scale
STROBE: Strengthening the Reporting of Observational Studies in Epidemiology
USA: United States of America
VAS: Visual Analogue Scale

## References

[1] Vos T, Barber RM, Bell B, Bertozzi-Villa A, Biryukov S, Bolliger I, Charlson F, Davis A, Degenhardt L, Dicker D (2015). Global, regional, and national incidence, prevalence, and years lived with disability for 301 acute and chronic diseases and injuries in 188 countries, 1990–2013: a systematic analysis for the global burden of disease study 2013. Lancet.

[2] Gaskin DJ, Richard P (2012). The economic costs of pain in the United States. J Pain.

[3] Laird RA, Gilbert J, Kent P, Keating JL (2014). Comparing lumbo-pelvic kinematics in people with and without back pain: a systematic review and meta-analysis. BMC Musculoskelet Disord.

[4] Chen S-PC, Samo DG, Chen EH, Crampton AR, Conrad KM, Egan L, Mitton J (1997). Reliability of three lumbar sagittal motion measurement methods: surface inclinometers. Journal of Occupational Environmental Medicine.

[5] Hemming R, Sheeran L, van Deursen R, Sparkes V (2018). Non-specific chronic low back pain: differences in spinal kinematics in subgroups during functional tasks. Eur Spine J.

[6] Laird RA, Keating JL, Kent P (2018). Subgroups of lumbo-pelvic flexion kinematics are present in people with and without persistent low back pain. BMC Musculoskelet Disord.

[7] O’Sullivan P (2005). Diagnosis and classification of chronic low back pain disorders: maladaptive movement and motor control impairments as underlying mechanism. Man Ther.

[8] Matheve T, Brumagne S, Demoulin C, Timmermans A (2018). Sensor-based postural feedback is more effective than conventional feedback to improve lumbopelvic movement control in patients with chronic low back pain: a randomised controlled trial. Journal of neuroengineering rehabilitation.

[9] Tsang SM, Szeto GP, Li LM, Wong DC, Yip MM, Lee RY (2017). The effects of bending speed on the lumbo-pelvic kinematics and movement pattern during forward bending in people with and without low back pain. BMC Musculoskelet Disord.

[10] Banos O, Moral-Munoz J, Diaz-Reyes I, Arroyo-Morales M, Damas M, Herrera-Viedma E, Hong C, Lee S, Pomares H, Rojas I (2015). mDurance: a novel mobile health system to support trunk endurance assessment. Sensors.

[11] Laird RA, Keating JL, Ussing K, Li P, Kent P (2019). Does movement matter in people with back pain? Investigating ‘atypical’lumbo-pelvic kinematics in people with and without back pain using wireless movement sensors. BMC Musculoskelet Disord.

[12] Mjøsund HL, Boyle E, Kjaer P, Mieritz RM, Skallgård T, Kent P (2017). Clinically acceptable agreement between the ViMove wireless motion sensor system and the Vicon motion capture system when measuring lumbar region inclination motion in the sagittal and coronal planes. BMC Musculoskelet Disord.

[13] Laird RA, Kent P, Keating JL (2016). How consistent are lordosis, range of movement and lumbo-pelvic rhythm in people with and without back pain?. BMC Musculoskelet Disord.

[14] Von Elm E, Altman DG, Egger M, Pocock SJ, Gøtzsche PC, Vandenbroucke JP (2007). The strengthening the reporting of observational studies in epidemiology (STROBE) statement: guidelines for reporting observational studies. Ann Intern Med.

[15] Cohen J (1992). A power primer. Psychol Bull.

[16] Cohen J. Statistical power analysis for the behavioral sciences: Routledge; 2013.

[17] StataCorp SL. College Station, Texas: StataCorp. Stata Statistical Software: Release. 2015:14.

[18] Charry E, Umer M, Taylor S. Design and validation of an ambulatory inertial system for 3-D measurements of low back movements. In: *2011 Seventh International Conference on Intelligent Sensors, Sensor Networks and Information Processing: 2011*: IEEE, vol. 2011. p. 58–63.

